# Establishing women’s cancer care services in a fragile, conflict and violence affected ecosystem in Africa

**DOI:** 10.3332/ecancer.2021.1231

**Published:** 2021-05-13

**Authors:** Groesbeck Preer Parham, Kabongo Mukuta Mathieu, Tankoy Gombo YouYou, Michael L Hicks, Ronda Henry-Tillman, Alex Mutombo, Mukanya Mpalata Anaclet, Mulumba Kapuku Sylvain, Leeya Pinder, Maya M Hicks, Louis Kanda, Mirielle Kanda

**Affiliations:** 1 Department of Obstetrics and Gynecology, University of North Carolina at Chapel Hill, 101 Manning Dr, Chapel Hill, NC 27514, USA; 2 Department of Obstetrics and Gynecology, University Teaching Hospital – Women and Newborn Hospital, 10101 Nationalist Way, Lusaka, Zambia; 3 Biamba Marie Mutombo Hospital, No. 9777, Boulevard Lumumba, Commune de Masina, Kinshasa, Democratic Republic of the Congo; 4 St Mary Mercy Cancer Center, 36475 Five Mile Rd, Livonia, MI 48154, USA; 5 St Joseph Mercy Oakland Cancer Center, 44405 Woodward Ave, Suite 202, Pontiac, MI 48324, USA; 6 McLaren Macomb Medical Center, 1000 Harrington Blvd, Mount Clemens, MI 48043, USA; 7 Winthrop P Rockefeller Cancer Institute, University of Arkansas for Medical Sciences, 4301 West Markham St, Slot #725, Little Rock, AR 72205, USA; 8 Department of Oncology, University of Washington, 1959 NE Pacific St, Seattle, WA 98195, USA; 9 Howard University College of Medicine, 520 W St NW, Washington, DC 20059, USA; 10 Dikembe Mutombo Foundation, 400 Interstate N Pkwy, Suite 1040, Atlanta, GA 30339, USA; a https://orcid.org/0000-0001-5922-5990; b https://orcid.org/0000-0002-1819-155X; c https://orcid.org/0000-0002-1782-9523; d https://orcid.org/0000-0002-8929-7810; e https://orcid.org/0000-0002-1993-3367

**Keywords:** fragile, conflict and violence affected societies, Democratic Republic of the Congo, cervical cancer, breast cancer, African cancer centre, Dikembe Mutombo

## Abstract

**Background:**

The majority of the world’s poorest women (income < $1.90/day) reside in fragile, conflict and violence (FCV)-affected countries, like the Democratic Republic of the Congo. Health services in these settings have traditionally focused on immediate relief efforts, communicable diseases and malnutrition. Recent data suggests there is need to widen the focus to include cancer, as its incidence and mortality rates are rising.

**Methods:**

Employing competency-based learning strategies, Congolese health professionals were trained to perform same-day cervical cancer screening and treatment of precancerous lesions of the cervix; same-day clinical breast examination and breast ultrasound diagnostics; surgical treatment of invasive cancers of the breast and cervix; and infusion of cytotoxic chemotherapy. Outpatient breast and cervical cancer care clinics, a chemotherapy suite and surgical theatres were outfitted with equipment and supplies.

**Results:**

Combining local and regional hands-on training seminars with wise infrastructure investments, a team of US and Zambian oncology experts successfully implemented a clinical service platform for women’s cancers in a private sector health facility in the Democratic Republic of the Congo.

**Conclusion:**

We forged a novel partnership between oncology health professionals from Africa and its Diaspora, international philanthropic organisations, a cancer medicine access initiative and an established African cancer centre to build women’s cancer services in a FVC-affected African setting.

## Introduction

Two billion people live in areas plagued by violence and conflict, among whom are the majority of the global poor (income < $1.90/day) and a third of the world’s women [1, 2]. Since 1990, there have been over 1,100 recorded conflicts across 102 countries, and the numbers are increasing [3]. While the effects of war and conflict are profoundly devastating, they can extend beyond the battlefield. By destabilising the political institutions that help to ensure basic human rights and freedoms [4], war and conflict create the social conditions (extreme poverty, food shortages, lack of safety, overcrowded and unsanitary living arrangements) that result in disease and death within the countries in which they occur [5]. The prioritisation of health issues in fragile, conflict and violence (FCV)-affected societies is largely dependent on international funding [6]. Recent data suggests there is need to include cancer as a priority health concern in these environments, as its incidence and mortality rates are rising. Of particular importance are cancers of the cervix and breast, because of their heavy burden among the world’s poorest women [7]. To be effective, cancer care services for women residing in these resources-constrained settings must address the following needs: (A) health promotion messages that consider the social, cultural and religious norms that impede the acceptance of allopathic cancer care; (B) clinical infrastructures for screening, early detection, diagnosis and treatment; (C) workforce development that includes oncology specialists, ancillary health professionals and technicians and (D) access to high-quality, low-cost cancer medicines and opioids.

Motivated by the need and our previous successes in establishing cancer care services for marginalised women in Africa and its Diaspora [8–13], we embarked on an initiative with the objective of developing and implementing a cervical and breast service platform in the Democratic Republic of the Congo (DRC), the largest fragile nation in Africa [14]. This report gives an overview of our approach to programme implementation in which workforce training was integrated with service delivery and clinical infrastructure development [9, 13]. Other manuscripts in this series provide a detailed and rigorous evaluation of the clinical outcomes of our activities [15–17].

## Background and rationale

The DRC is the second largest country on the African continent and major African continental crossroad being bordered by Rwanda, Uganda, Burundi and Tanzania to the east, Central African Republic and South Sudan to the north, Angola and Zambia to the south and Republic of Congo to the west. Formally established as a Belgian colony in 1908, the DRC gained independence from Belgium in 1960, became Zaire in 1972 and formally DRC in 1997. The population of 84 million people is now host to over 200 different ethnic groups and distinct languages with Lingala, Kingwana, Kikongo, Tshiluba and French considered the national languages [18]. The DRC is fraught with centuries of political destabilisation, armed clashes, internal wars and human rights violations. Much of the conflict has been centred around control of its vast reserves of gold, diamonds, tungsten, uranium, oil and coltan – an ore used in laptops and mobile phones [19]. The combination of conflict, economic plunder by multinational corporations and internal corruption has had a devastating effect on the living conditions of its people and stability of its public institutions, including the collapse of the healthcare system. According to the World Bank, approximately 70% of the DRC’s 84 million inhabitants survive on less than $1.90/day, ranking it 179th of 189 countries by the Human Development Index [20]. Over 40% (33 million) of its people have no access to quality water, despite the fact that over 50% of Africa’s water reserves are located in the DRC. Less than 10% of the population has access to electricity. Over 70% of its people lack adequate food, including children under age 5, 50% of whom are affected by chronic malnutrition and 1.3 million by severe acute malnutrition [21]. Only 35% of children aged 12–23 months are fully vaccinated before their first birthday, with 20% receiving no vaccinations at all. The country suffers from a severe shortage and inequitable distribution of healthcare personnel. There are 0.28 physicians and 1.91 nurses and midwives per 10,000 population, with the northern rural areas of the country having the fewest health workers, particularly physicians (1.1%–2.6%) [22]. Few students from any of the 39 nationally recognised medical schools have the opportunity to spend the required years in clinical rotations due to a lack of participating hospitals. The overall result is an education of doubtful quality [23, 24]. Residency training is rare with approximately 2.5% of the rural-school graduates and 15.2% of urban-school graduates entering formal residency programmes. Deficiencies in the public health system are central to the recent outbreaks of measles, circulating vaccine-derived polioviruses type 2 and cholera in 2019, and Ebola a year earlier [25, 26].

Against this backdrop of economic deprivation, political instability and infectious disease epidemics, a cancer crisis is also unfolding in the DRC. Cancer is among the leading causes of death worldwide. In 2018, there were 18.1 million new cancer cases and 9.5 million cancer-related deaths globally, far exceeding the number of deaths from human immunodeficiency virus/AIDS, malaria and tuberculosis combined [27]. World Health Organization (WHO) predicts that by 2040, the annual number of new cancer cases in the world will rise to 29.5 million and cancer-related deaths to 16.4 million [28]. While an estimated 60% of cancer cases and 75% of deaths will occur in low- and middle-income countries [29], only 5% of global spending [30] on cancer is directed to these countries. Two cancers of particular importance are those originating in the cervix and breast, as their incidence rates are rising, mortality rates are among the highest in the world and together constitute half of all new cases of cancer in women in the African region [31, 32]. It is predicted that without interventions, the annual number of new cases and related deaths from these two cancers will more than double by 2040 [32]. Among all new adult malignancies diagnosed in the DRC in 2018, cancers of the breast (12.6%) and cervix (11.8%) were the most common, together accounting for almost half of the 27,000 cancers diagnosed in females [33]. This is almost certainly a major underestimation as case ascertainment is poor in urban settings and non-existent in most of the rural areas of the country. Although cervical cancer is the number one cause of cancer-related death among Congolese adults [33], public awareness about the disease remains extremely low. In a recent cross-sectional study of 524 women aged 16–78 years (median age: 28 years), in the capital city of Kinshasa, <20% of women interviewed knew the cause of cervical cancer or how it could be prevented or treated; <10% had ever had a Pap (cytology) smear and >70% used plants or chemicals for vaginal care [34].

With the common goal of improving healthcare in Africa, a partnership was formed between the Dikembe Mutombo Foundation (DMF) [35] and the Friends of Africa, Inc. (FOA) [36], both US 501(c)3 organisations. Founded and led by the US National Basketball Association legend Dikembe Mutombo, originally from the DRC, the DMF is dedicated to improving the health, education and quality of life for the people of the DRC. The organisation strives to accomplish its mission by promoting primary health care and disease prevention, health policy, research and increased access to health care education. In 2007, to help realise its goals, the DMF constructed a privately-owned, 150-bed primary care hospital – Biamba Marie Mutombo Hospital (BMMH) – in central Kinshasa. The FOA is an organisation primarily composed of US trained and board-certified surgical oncologists committed to reducing cervical and breast cancer mortality in Africa and its Diaspora. Founded in 1989, the FOA (1) develops training programmes and clinical services for the early detection of women’s cancers; (2) sponsors onsite, hands-on surgical oncology education seminars; (3) promotes local ownership of medical clinics and supply chains and (4) supports practical and relevant scientific research activities.

Our primary objective was to increase access to women’s cancer (cervix and breast) services in the DRC by building a contextually-appropriate and sustainable diagnostic and therapeutic service platform.

## Methods

### Situational assessment

#### Consensus meeting

The first step in assessing the local conditions and circumstances was to convene a broad cross section of stakeholders for a Consensus Meeting. In March 2016, key Congolese stakeholders from the government, civil society, private and public health sectors and academia were convened over a 3-day period in the capital city of Kinshasa. The format of the meeting consisted of formal presentations of peer-reviewed, gray literature and unpublished data on the following topics: (1) epidemiology of women’s cancers in the African region with a focus on the DRC, [33, 34, 37–45] and (2) current cancer care workforce capacity, clinical services and infrastructure in the DRC [46, 47] (Table 1).

#### Hospital site visit

Following the Consensus Meeting, a site visit was conducted at BMMH by a visiting US gynaecologic oncologist (Groesbeck Parham). Discussions were held with hospital administrators, clinicians (gynaecologists, surgeons, nurses, anaesthesiologists), clinical managers, procurement specialists, data managers and DMF leadership. An assessment was made of the physical plant, clinical capacity, infrastructure and interest among staff in participating in a women’s cancer care initiative.

### Implementation strategy

Informed by the findings from the situational assessment, a team of US and Zambian surgical and clinical oncologists and nurse specialists was tasked by the FOA to design an implementation strategy that encompassed training a local oncology workforce, developing clinical services and building a clinical infrastructure for women’s cancer care services.

## Results

### Situational assessment

Stakeholders attending the Consensus Meeting listed the following as major barriers to the implementation of women’s cancer care services in the DRC: (1) low levels of cancer awareness among health professionals and the general population; (2) lack of trained oncology human resources of all types, including ancillary personnel; (3) lack of financing and unreliable systems for the procurement, storage and dispersal of equipment and supplies; (4) poor access to affordable cancer medicines and (5) properly outfitted clinics and surgical theatres (Table 1).

### Implementation strategy

#### Workforce capacity building

The above findings, coupled with a wide variation in the backgrounds and clinical experiences of the designated BMMH staff-trainees, prompted the FOA team to adopt competency-based training enfolded into a matrix of self-directed learning and bedside mentoring as the strategy for workforce capacity building. Mastery of the various clinical and surgical procedures was facilitated by using the following skills-building techniques: deconstruction (each procedure was broken down into its subcomponents); high volume repetition (repeating the same clinical procedures over short time intervals); pre- and post-case review and mental narration (imaginary enactment of clinical procedures). South-south professional exchange programmes were also felt to represent potential opportunities for skill building. Onsite mentoring was led by the visiting FOA oncology team on an intermittent basis, as conditions permitted.

#### Clinical services

Given the low socioeconomic status of the target population and their lack of access to cancer prevention and treatment services, the clinical services deemed to be of greatest value were the following: (1) cervical cancer screening using visual inspection with acetic acid (VIA) and digital cervicography; (2) treatment of cervical cancer precursors using thermal ablation and large loop excision of the transformation zone (LLETZ); (3) evaluation of symptomatic women with clinical breast exam, breast ultrasound and ultrasound-guided core needle biopsy/fine needle aspiration; (4) surgical treatment of cervical and breast cancers; (5) breast oncoplastic procedures; (6) palliative surgery and (7) infusion of chemotherapy for advanced cervical and breast cancers (Table 1).

#### Infrastructure investments

Investments were made in outpatient clinics and surgical theatres to ensure that the trainers and trainees had well-equipped clinical facilities for both teaching and clinical care, respectively. Dedicated space for the new clinical services was assigned by the hospital administration, after which it was remodelled and furnished with the necessary equipment, supplies and furniture (Table 2).

#### Timeline

The initial plan was to implement workforce training, clinical service and infrastructure development through quarterly visits over a 12-month period. The intended timeline was significantly altered from 12 to 24 months (January 2017–December 2019) due to travel restrictions related to multiple episodes of political unrest within the DRC and Ebola outbreaks. Mentorship was continued in between visits through electronic communication [9].

## Clinical outcomes

### Cervical cancer

Following approval by the DRC Ministry of Health, the DMF and FOA, in collaboration with the United Nations Population Fund, co-sponsored a cervical cancer awareness campaign in Kinshasa (May–June 2016). Awareness activities, led by teams of nurses and doctors from the BMMH, were comprised of television and radio announcements and talk shows, combined with community-based educational sessions at large public gathering sites (markets and religious services) throughout the city. Immediately following the campaign, a small team of BMMH staff doctors and nurses attended a hands-on cervical cancer prevention educational seminar at the University Teaching Hospital in Lusaka, Zambia (20 June–1 July 2016), where they were trained to perform ‘single-visit cervical cancer screening and treatment’ procedures using VIA, digital cervicography, thermal ablation and LLETZ [13]. Upon completion of their training, they returned to the DRC and established the BMMH Cervical Cancer Early Detection and Treatment Clinic, based on the Zambian model [13]. A cervical cancer ‘screen and treat’ camp was announced on radio and television then held at the BMMH (13—18 July 2016). During the week-long event, over 8,000 women presented for cervical cancer screening services. Screening was conducted by the newly trained BMMH team during the week-long camp, under the direct supervision of cervical cancer prevention nurse-trainers from Zambia. Clients found to have precancerous cervical lesions were offered same-day treatment using either thermal ablation or LLETZ by the BMMH staff. Those whose cervical lesions were grossly suspicious for invasive cancer were evaluated with punch biopsy. If pathologically confirmed to have early-stage invasive cancer, they were counselled and offered surgery by the visiting team of US surgical oncologists (gynaecologic oncologists), during which time BMMH staff gynaecologists were taught how to perform radical abdominal hysterectomy, bilateral pelvic lymphadenectomy [9]. All women who underwent screening were also asked by screening nurses if they had breast symptoms. Those who did were referred to the newly opened BMMH Breast Clinic for further evaluation.

**Figure 1.**
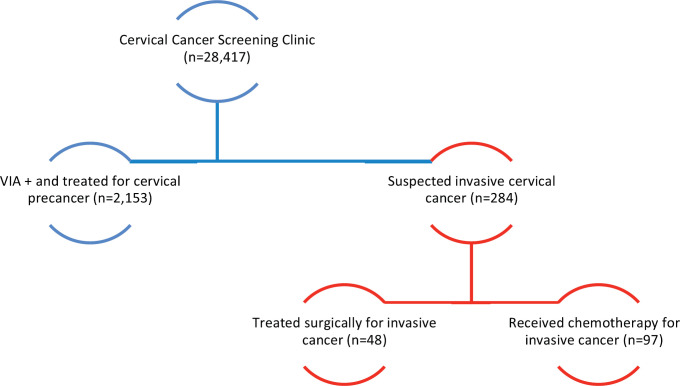
Cervical cancer programme outcomes.

As of January 2020, 28,417 women have been screened for cervical cancer, of which 2,153 (1 of every 13 screened) were found to have cervical abnormalities suggestive of cervical pre-cancer. More than 90% were treated on the same day with either thermal ablation or LLETZ. Invasive cervical cancer was pathologically confirmed in 284 (1%) of the screened population (Figure 1). of which 48 (17%) patients were early stage and operable. Patients with advanced stage invasive cancers were referred to the national cancer centre in Zambia for radiation therapy. Those who could not afford radiation therapy in Zambia were put on a palliative care regimen, as there were no chemotherapy or radiation therapy facilities in the DRC during the time of programme implementation (Figure 1).

### Breast cancer

Following the initial cervical cancer screening camp in July 2016, four additional visits were made to the DRC by the FOA team of mentors from January 2017–December 2019. During these visits, they were joined by a US breast surgical oncologist (Ronda Henry-Tillman) who implemented a hands-on training curriculum for BMMH staff general surgeons and nurses, designed to teach clinical breast examination, ultrasound imaging of the breast and axilla ultrasound-guided core needle biopsies and fine need aspirations. A surgical training curriculum for staff general surgeons was initiated by the US breast surgical oncologist with the goal of building their capacity to perform breast lumpectomy, modified radical mastectomy, axillary node dissections, breast oncoplastic procedures and palliative surgery. The curriculum for teaching breast diagnostic and surgical procedures was previously developed and implemented in Zambia [12].

**Figure 2.**
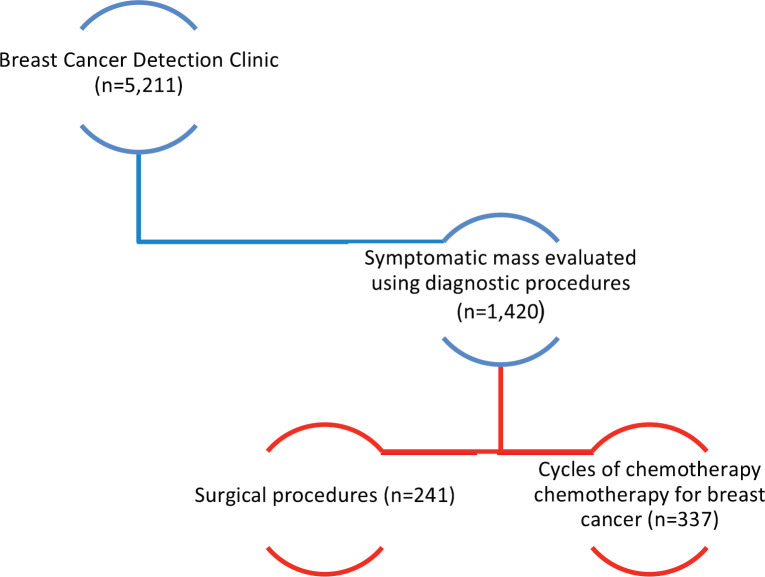
Breast cancer programme outcomes.

As of December 2019, 5,211 women have been evaluated using clinical breast examination and breast ultrasound. The vast majority presented with symptoms, elicited by nurses during cervical cancer screening. Clinical abnormalities were detected in 1,420 (27%) women, of which 516 (36%) met the criteria for ultrasound-guided core needle biopsy. Pathology reports were available on 368 (71%), of which 164 (44%) were malignant and 204 (55%) benign. Approximately 90% of the breast cancers were advanced stage (stage 3 or 4) (Figure 2). A total of 241 women have undergone surgery, consisting of 183 breast lumpectomies, 58 modified radical mastectomies and 48 axillary lymph node dissections (LND) (Figure 2). The specifics of this programme and outcomes are described in the accompanying breast manuscript [17].

### Chemotherapy

Some of the chemotherapeutic agents indicated for the treatment of breast cancer were available for purchase from local private vendors in Kinshasa; however, their prices made them unaffordable for the vast majority of patients. Neither was there a mechanism for verifying their quality. Access to cytotoxic cancer medicines at a discounted price became available through a partnership with the Bioventures for Global Health (BVGH) *African Access Initiative* [48], a nonprofit organisation that expands access to cancer medicines and technologies in Africa through a sustainable access model. To ensure the quality of the cancer drugs, only companies whose products are United States Food and Drug Administration-approved are invited to participate. The partnership with BVGH created opportunities for the development of a chemotherapy unit at BMMH. To satisfy the need for training, an intense, hands-on, short-course (6 weeks) in chemotherapy administration was developed at Zambia’s national cancer centre – Cancer Diseases Hospital – tailored for BMMH health professionals. Under this curriculum two BMMH general physicians and three nurses were trained to safely and effectively administer chemotherapy for breast and cervical cancer, following strict protocols. Upon course completion, the trainees returned to the DRC and established the BMMH Chemotherapy Infusion Unit in June 2019. Guided by clinical oncologists from Zambia’s national cancer centre, the infrastructure for the chemotherapy was established. Oversight and quality assurance were provided through a combination of onsite visits and teleconferencing with a senior chemotherapy nurse and clinical oncologist from the Zambian cancer centre. This experience is described in detail in the chemotherapy manuscript in this series [16]. Since forming a partnership with BVGH, over 100 breast and cervical patients have been safely treated with chemotherapy, having received a total of 377 cycles of cytotoxic agents. This model of purchasing chemotherapy drugs at a reduced cost is one of the only options for accessing essential cancer medicines, irrespective of whether they exist on national formularies. For more details, refer to the chemotherapy manuscript in this series [16].

### Human resources

A wide variety of 21 BMMH staff members were trained during the course of the initiative (Table 2).

## Discussion

We report one of the few real-world efforts to implement detection and treatment services for cervical and breast cancer in a fragile nation torn by war, outbreaks of infectious diseases, political unrest and devastating poverty.

Using a unique matrix of self-directed learning, onsite and regional hands-on training seminars, south-south professional exchange programmes and teleconferencing, Congolese-led women’s cancer services were established in a private healthcare facility in Kinshasa. Skills transfer was facilitated through a process of clinical and surgical bedside mentoring, rendered by a close-knit team of highly skilled university-based US and Zambian oncologists and Zambian nurse-specialists who had a history of working together in resource-constrained African environments. Through wise investments, the physical and clinical infrastructure needed to support the newly trained workforce was simultaneously established. In between onsite visits, modern forms of telecommunication (e.g., Zoom teleconferencing) were used to facilitate continuing medical education. Central to the success of the effort was the support of the hospital’s administrative staff and the sponsoring organisation (DMF), coupled with the leadership, clinical knowledge and logistical guidance provided by local professional colleagues.

### Meaning and context

Fragility and violent conflict are on the rise worldwide. When protracted, their consequences extend beyond the war-wounded, impacting the health of entire communities. By 2030, up to two-thirds of the global extreme poor will live in societies affected by fragility, conflict and violence [1]. Noncommunicable diseases, including cancer, are increasing in these settings [49, 50], and women are disproportionately impacted. Of the 2 million annual new cases of cervical and breast cancer and 800,000 annual cancer-related deaths that occur in the world, a significant percentage are in women who live in the fragile and conflict ecosystems of sub-Saharan Africa [1], where opportunities for cancer care are limited [51, 52].

Building the pathway for cancer care in severely resource-constrained settings is a difficult task, as it involves the integration of a cancer care platforms into healthcare systems whose supportive services (blood banking, laboratory, procurement, pathology, pharmacy, etc.) are often quite meagre. Yet, these are the circumstances under which one must operate when faced with the task of implementing cancer care services in the world’s poorest nations. When working in settings that are severely resource-constrained, a key ethical question that must be addressed is whether attempts should be made to implement only certain parts of the cancer care pathway if the system cannot be implemented in its entirety. In our particular situation, the outstanding question was whether cancer detection, diagnostic and surgical services should be implemented in the absence of radiation therapy services, given the likelihood of uncovering large numbers of cases of advanced disease that would require these adjunctive therapeutic modalities. When posed to key local stakeholders and organisations in attendance at the Consensus Meeting, the consensus opinion was to begin, immediately, by training the workforce and building the clinical services and infrastructure needed for delivery of early detection and surgical treatment services. The underlying rationale was that the present inability to treat all cancer cases should not impede efforts to *prevent* those cases that can be prevented (e.g. ablation of precancerous conditions of the cervix), *treat* those cases that can be treated (e.g. early-stage cancers that can be cured with surgery alone) and *relieve suffering* in the others. These recommendations were in line with the humanitarian principles embedded in the ‘Four Pillars of Oncology’: prevent all cancers that can be prevented; treat all cancers that can be treated; cure all cancers that can be cured; provide palliation whenever palliation is required [53]. There was also general consensus that efforts should be pursued to fill the remaining gaps in the clinical care pathway by addressing the barriers and establishing access.

Motivated by a commitment to actively engage the problem by implementing contextually-appropriate and evidence-based solutions, and to learn on the go, we started with the resources that were available. We made frequent use of the various sets of resource-stratified management guidelines for cervical and breast cancer developed by the WHO, Breast Health Global Initiative, National Comprehensive Cancer Network and American Society of Clinical Oncology [54–57]. Similar sets of rigorously examined strategies for implementation in resource-constrained settings are lacking, particularly as it pertains to FCV settings. In developing an implementation strategy, we relied on our understanding of the natural history of the diseases, accumulated experience working as clinicians in low-income African countries and underserved communities in high-income countries and a clear-headed assessment of what could be done safely, effectively and in a sustainable fashion. In-country and regional (south-south) trainings were emphasised, as opposed to training in high-income countries, which can lead to translation difficulties because of vast contextual differences. The decision to execute training and diagnostic and treatment services within a private versus public sector facility served to circumvent some of the logistical problems we might have confronted in the public sector, such as lack of personal security, bureaucratic inefficiencies, unreliable procurement systems and the potential for politicisation and militarisation of health services.

### Limitations

Cancer care as a human right is in accordance with human rights instruments adopted by the United Nations Human Rights Council [58]. We recognise the tension between a private sector model that rests on a fee-for-service platform in a setting in which there are no government-supported social insurance schemes. However, even when services appear to be ‘free’, they are in fact subsidised or paid for by external or even internal philanthropy, e.g., substantial fees paid by private patients to cover the expenses of those who are unable to pay. The ultimate answer is a well-financed Universal Health Care insurance scheme funded by local governments, as outlined in the United Nations 2030 Agenda for Sustainable Development [59, 60]. By attaching cancer control services to a fee-for-service healthcare facility, fewer people will be able to afford them but the chances they will be sustained may be increased, particularly in fragile and conflict-affected settings.

### Challenges and solutions

Some of the challenges encountered and solutions derived during the course of our work were as follows:

#### Supply/demand imbalances for screening services

In order to increase the availability and accessibility of cancer detection services, quarterly week-long mobile outreach cervical cancer early detection camps were sponsored by the BMMH. To date, six such outreach camps have been held, all in densely populated areas around Kinshasa, led by hospital nursing staff, through which over 5,000 women have been screened for cervical cancer. Three of the outreach camps were mentored, onsite, by visiting Zambian nurse-trainers.

#### High cost of pathology services

The costs of pathology services were unaffordable for most patients, resulting in significant delays, and impacting timely treatment. As a short-term solution, we negotiated reduced fees with private sector pathologists in Kinshasa, however, most patients still had difficulty paying the residual costs. These circumstances helped to motivate the formation of a partnership between the DMF and American Society of Clinical Pathology, resulting in a venture to build chemistry and pathology laboratory infrastructure at the BMMH. All equipment has been purchased, installed and a local pathologist hired to lead the programme. Expectations are that the laboratory will be fully operational in April 2021.

#### Access to cancer medicines

The World Health Organization has an Essential Medicines List that contains several chemotherapeutic and palliative agents used in the treatment and palliation of breast and cervical cancer [61]. Other than tamoxifen, these medicines are difficult to acquire or are unaffordable for governments or patients in many sub-Saharan African countries. Purchasing chemotherapy, even at low cost, inevitably leads to resource limitations in that it is not sustainable. However, if properly managed, it can be creatively leveraged to generate profit that can be used to support future purchases. We were able to partner with an international non-profit global health organisation (BVGH) that facilitates access to selected cancer medicines at a reduced cost [48].

#### Lack of radiation therapy services

Radiation therapy is an essential pillar in the treatment of cervical and breast cancer. Early-stage cervical cancers with low disease volume can often be cured with surgery alone. Others require adjunctive treatment with radiation. Radiation therapy is also essential in the management of breast cancer patients who are treated with conservation therapy as well as those with advanced disease. We were aware that we would encounter patients that could not be optimally treated because of the lack of radiation therapy. This problem is not just limited to the DRC but across the region as only 23 of 52 African countries are known to have radiation therapy facilities [62]. This was the central dilemma in our decision to start with what was available instead of waiting until all components in the cancer care pathway were in place. However, not to begin with the resources we had at hand would lead to continued missed opportunities to prevent cancers and cure the patients that we could, and maybe prolong the lives of others. Towards that end, present discussions are underway to build a collaborative relationship between BMMH and a new privately-owned radiation therapy centre in Kinshasa.

### Implications for the future

Limited, one-time investments such as ours can be very beneficial in that they can provide the capital outlay for training and infrastructure development that can be continually realised. The presence of women’s cancer care at BMMH can now be used to offset expenses by offering services at affordable but profitable fees. It can also be presented as a model of cancer care to investors interested in promoting the growth of for-profit health enterprises in low-income nations, particularly those affected by conflict and violence. Services can be leveraged to advocate for government-led national health promotion and awareness campaigns for cervical and breast cancer control; public sector breast and cervical cancer early detection and treatment services; national Human Papilloma Virus vaccination programmes for adolescent girls; expansion of national laboratory and pathology services; public sector radiation therapy services; partnerships with pharmaceutical firms to help ensure access to essential cancer medicines and the development of a national strategic plan for cancer control.

Private enterprises can help break the cycle of fragility, conflict and poverty. Businesses create jobs and income and support livelihoods, and in doing so can contribute to social cohesion and stability. Private investments in cancer care in resource-constrained environments deserve further consideration [63]. This marriage may have the best chance of success in a private, non-profit institution like the BMMH where the ultimate goal is to balance the economic capacity of the community with the financial resources required to deliver a high quality of care and ensure institutional sustainability.

## Conclusion

Using a novel partnership consisting of oncology health professionals from Africa and its Diaspora, international philanthropic organisations, a cancer medicine access initiative and an established African cancer centre, we successfully trained the workforce and built the clinical services and infrastructure for women’s cancer care services in a private sector health facility in the DRC.

## List of abbreviations

BMMH, Biamba Marie Mutombo Hospital; BVGH, Bioventures for Global Health; DMF, Dikembe Mutombo Foundation; DRC, Democratic Republic of the Congo; FCV, Fragile, Conflict and Violence; FOA, Friends of Africa, Inc.; HPV, Human papilloma virus; LLETZ, Large loop excision of the transformation zone; LND, Lymph node dissection; VIA, Visual inspection with acetic acid; WHO, World Health Organization.

## Conflicts of interest

None of the authors declare any conflicts of interest.

## Funding statement

Funding for the initiative was provided by a generous grant from the Howard G. Buffett Foundation [34], whose mission is to catalyse transformational change to improve the standard of living and quality of life, particularly for the world’s most impoverished and marginalised populations.

## Figures and Tables

**Table 1.** Assessment and training.

Activities	Outcomes
Rapid situational assessment of the status of women’s cancer services in the DRC	Consensus Meeting of key stakeholders from government, civil society, business and academia Onsite assessment of activities and human resources of BMMH Gaps, needs and solutions identified
Awareness activities – Cervical/breast cancer	Cervical and breast cancer public awareness campaigns in DRC (Kinshasa) Television and radio announcements Community-based awareness events
Training activities – Cervical cancer	Hands-on training for cervical cancer prevention in Zambia and DRC BMMH staff doctors and nurses trained to perform ‘single-visit cervical cancer screening and treatment’ by visiting Zambian nurse experts On-site surgical training in DRC BMMH staff gynaecologists trained to perform radical abdominal hysterectomy and bilateral pelvic lymphadenectomy by visiting US gynaecologic oncologists
Training activities – Breast cancer	On-site clinical and surgical training in DRC BMMH staff general surgeons and nurses trained to evaluate breast masses and axilla with ultrasound and ultrasound-guided core-needle biopsy/fine needle aspiration by visiting US breast oncologist BMMH staff general surgeons trained to perform lumpectomy and modified radical mastectomy and axillary LND^a^, palliative and breast oncoplastic procedures by visiting US breast oncologist
Training activities – Chemotherapy	Hands-on short course in chemotherapy administration in Zambia and DRC BMMH staff physicians trained in principles and practice of safe administration of chemotherapy for cervical and breast cancer by clinical oncologists at Zambia’s national cancer centre

*^a^LND, Lymph node dissection

**Table 2.** Infrastructure investments.

Types	• Details
Health services	Cervical cancer prevention clinic Single-visit screening and treatment services Cervical cancer surgical unit Surgical management of early-stage cervical cancer Breast cancer clinic Clinical breast examination, diagnostic ultrasound, core-needle biopsy Breast cancer surgical unit Surgical management of breast cancer, including oncoplastic procedures Chemotherapy suite Administration of chemotherapeutic agents to treat breast and cervical cancer
Human resources	Cervical cancer ‘screen and treat’ providers (5) Gynaecologists trained to perform radical abdominal hysterectomy and lymphadenectomy (3) General surgeons trained to perform breast conservation and palliative surgery, and oncoplastic procedures (2) General surgeons (2) general physicians (2) and nurses (3) trained to perform breast diagnostics General physicians (2) trained to administer chemotherapy Procurement officer (1) Data manager (1)
Equipment and supplies	Procured clinic and operating room equipment and supplies (ultrasound machines, examination tables, core needle biopsy, surgical instruments, suture material, gynae examination equipment, thermal ablation and loop electrosurgical excision procedure /LLETZ, digital cameras, computers, smart phones, storage facilities for chemotherapy drugs, personal protective equipment, chemotherapy hood)
